# Artificial intelligence in the analysis of emotions of nursing students undergoing clinical simulation

**DOI:** 10.1590/0034-7167-2021-0909

**Published:** 2023-04-14

**Authors:** Casandra Genoveva Rosales Martins Ponce de Leon, Leandro Yukio Mano, Danielle da Silva Fernandes, Rayanne Augusta Parente Paula, Guilherme da Costa Brasil, Laiane Medeiros Ribeiro

**Affiliations:** I Universidade de Brasília. Brasília, Distrito Federal, Brazil; II Universidade Estadual do Rio de Janeiro. Rio de Janeiro, Rio de Janeiro, Brazil; III Secretaria de Saúde do Distrito Federal. Brasília, Distrito Federal, Brazil; IV Centro Universitário do Distrito Federal. Brasília, Distrito Federal, Brazil

**Keywords:** Nursing, Nursing Students, Simulation, Training with High Fidelity Simulation, Emotions, Enfermería, Estudiantes de Enfermería, Simulación, Entrenamiento con Simulación de Alta Fidelidad, Emociones, Enfermagem, Estudantes de Enfermagem, Simulação, Treinamento com Simulação de Alta Fidelidade, Emoções

## Abstract

**Objective:**

to assess nursing students’ emotions undergoing maternal-child clinical simulation.

**Methods:**

an observational study, carried out between June and July 2019. The Focus Group technique was used, with 28 nursing students, randomly distributed into three groups, with qualitative (Bardin technique) and quantitative data (Artificial Intelligence) analysis, to analyze emotions through facial expressions, tone of voice and description of speeches.

**Results:**

we defined two categories: “It was not easy, it was very stressful”; and “Very valuable experience”. In Artificial Intelligence, emotional distribution between face, voice and speech revealed a prevalence of negative valence, medium-high degree of passivity, medium power to control the situation and medium-high degree of obstruction in task accomplishment.

**Final considerations:**

this study revealed an oscillation between positive and negative emotions, and shows to the importance of recognizing them in the teaching-learning process in mother-child simulation.

## RESUMO

**Objetivo:**

avaliar as emoções dos estudantes de enfermagem na vivência da simulação clínica materno-infantil.

**Métodos:**

estudo observacional, realizado entre junho e julho de 2019. Utilizada a técnica de Grupo Focal, com 28 estudantes de enfermagem, distribuídos aleatoriamente em três grupos, com análise dos dados qualitativa (técnica de Bardin) e quantitativa (Inteligência Artificial), para a análise das emoções através das expressões faciais, tom de voz e descrição das falas.

**Resultados:**

definiram-se duas categorias: “Não foi fácil, foi muito estressante”; e “Experiência muito valiosa”. Na Inteligência Artificial, a distribuição emocional entre face, voz e fala revelou prevalência da valência negativa, médio-alto grau de passividade, médio poder de controle da situação e médio-alto grau de obstrução na realização da tarefa.

**Considerações finais:**

este estudo revelou oscilação entre emoções positivas e negativas, e aponta para a importância de reconhecê-las no processo de ensino-aprendizagem na simulação materno-infantil.

## RESUMEN

**Objetivo:**

evaluar las emociones de estudiantes de enfermería en la experiencia de simulación clínica materno-infantil.

**Métodos:**

estudio observacional, realizado entre junio y julio de 2019. Se utilizó la técnica de Grupo Focal, con 28 estudiantes de enfermería, distribuidos aleatoriamente en tres grupos, con análisis de datos cualitativos (técnica de Bardin) y datos cuantitativos (Inteligencia Artificial), para el análisis de emociones a través de expresiones faciales, tono de voz y descripción de discursos.

**Resultados:**

definimos dos categorías: “No fue fácil, fue muy estresante”; y “Experiencia muy valiosa”. En la Inteligencia Artificial, la distribución emocional entre rostro, voz y habla reveló un predominio de valencia negativa, grado medio-alto de pasividad, poder medio para controlar la situación y grado medio-alto de obstrucción en la realización de la tarea.

**Consideraciones finales:**

este estudio reveló una oscilación entre emociones positivas y negativas, y apunta a la importancia de reconocerlas en el proceso de enseñanza-aprendizaje en la simulación madre-hijo.

## INTRODUCTION

Emotions are a complex reaction that involves the individuals’ entire organism, having a direct relationship with their needs, goals, values and well-being. In the general study of emotions and their interrelationships, different elements are considered and bring up the discussion about concepts and peculiarities, when one wants to determine their meaning^(1-2)^.

Over the years, several emotional models have been proposed, including two main strands: continuous and categorical models^(3-6)^. In continuous models, the main characteristic is the similarity in the way people show emotions observed in everyday life^(1)^, allowing them to be easily associated with facial expressions, as they imply changes that follow users’ emotional experience^(1-2)^.

In categorical models, Ekman (1973 and 2006) proposes a discrete pattern of basic emotions: happiness, disgust, fear, anger, surprise and sadness. The author argues that such emotions are those universally recognized, regardless of language or culture involved in communication^(4,6)^.

In the context of educational training, some studies questioned whether students’ emotions can impair learning^(7)^ in undergraduate nursing^(8-9)^, in multidisciplinary training contexts^(10)^ and in the anesthesiologist nursing program^(11)^. However, although they have assessed students’ satisfaction, self-confidence and stress in these different contexts, such researches have not deepened, based on a theoretical-methodological framework, students’ emotional behavior inserted in the teaching-learning process^(12-14)^_,_ even though negative feelings and emotional or psychological insecurity arose during simulation^(15)^. Still, predominantly, research points to using the method as beneficial for learning new skills (technical and non-technical), compared to other methodologies^(13,16)^.

In the maternal-child literature, no studies were identified demonstrating the interrelationship between clinical simulation experience emotions and learning. However, we identified two systematic review studies with different approaches: the first, in 2015, aimed to identify the adequacy and meaning of mother-child simulation-based learning for undergraduate nursing students, veterans or freshmen, in educational settings, for curriculum decision-making^(17)^; the second sought to identify the educational effectiveness of high-fidelity simulation, compared to none or a low-fidelity simulation, in neonatal resuscitation training^(16)^.

Given this gap, and considering the need to develop simulated practices in the nursing training process that are increasingly effective and emotionally and psychologically safe, for the current generation of university students, it was proposed to assess nursing students’ emotions in the maternal-child clinical simulation experience.

This work is justified because clinical simulation is increasingly inserted in undergraduate nursing curricula, helping students to make decisions in the course’s practical scenarios. According to the Brazilian National Curriculum Guidelines, nursing courses are structured, for the most part, with extensive course load, focusing predominantly on adults, and a subject on maternal-child health with reduced hours, offered almost at the end of the course. Nursing students are not familiar with this population (pregnant, puerperal, newborn, infant, child and adolescent), and it is essential to study their emotions in this context, for professors to work on empowering these students who will soon be inserted in the practical field. Emotions continually influence the relationship with co-workers, patients and family members; therefore, it is essential that students be able to access their own emotions and gain more security when dealing with intercurrences in the professional environment and making the best choices. For professors, in turn, it is relevant to understand the relationship between emotions and the learning of maternal-child nursing skills and competences.

The following guiding question emerged: what emotions did nursing students express when experiencing the maternal-child clinical simulation and what is the relationship between these emotions and learning?

A way to analyze human behavior, under these circumstances, was through Artificial Intelligence (AI), when examining individuals’ facial expressions, tone of voice and speech content, revealing the emotions that individuals often seek to protect. AI analysis in maternal-child nursing, through the use of clinical simulation, is unprecedented. Other studies have worked with the technique, but in the context of elder health applied to the household^(18)^ and adult health in practical scenarios^(19)^. The importance of using AI lies in the ability of this technology to analyze human emotions objectively, through the images (faces), voice spectra (recording) and speech content (transcription of participants’ speeches) identified.

Thus, the impact of this analysis for teaching maternal-child nursing, through the use of clinical simulation, is related to the imminent reflection of the teaching process, considering students’ emotions (negative valence or positive valence) and how these impact cognitive load, which, in turn, influences working memory, learning (long-term memory)^(7)^ and individuals’ actions.

## OBJECTIVE

To assess nursing students’ emotions undergoing maternal-child clinical simulation

## METHODS

### Ethical aspects

This article presents data from a thesis entitled “*Percepção dos students sobre a experiência da simulação clínica no ensino de enfermagem materno-childil: inter-relação entre as emoções e aprendizagem*”, developed together with the Graduate Program in Nursing at the Universidade de Brasília (PPGEnf/UnB).

The study was approved by the Research Ethics Committee of the Faculty of Health Sciences at UnB, and followed the guidelines and standards of the Ministry of Health Resolution 466 of December 12, 2012. To ensure participant anonymity, we adopted an alphanumeric system: first, we used the order of the day in which the collections took place; second, we use a letter (S for students) and an Arabic numeral indicating the order of transcription of the speeches, creating an acronym D1S1 and so on.

### Study design

This is an observational qualitative study. This research followed the recommended guidelines for qualitative studies through COnsolidated criteria for REporting Qualitative research (COREQ)^(20)^.

### Theoretical-methodological framework

With AI, Scherer’s Circumplex Model^(1)^ was chosen as the theoretical-methodological framework to analyze students’ emotional behavior.

**Figure 1:**
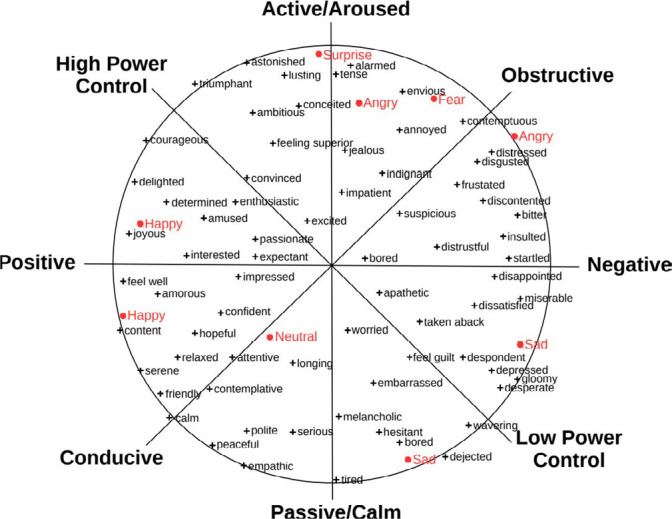
Scherer’s Circumplex Model^(1)^

The Circumplex Model^(1)^ (Figure 1) shows that all basic emotions are in a continuous two-dimensional space, whose dimensions are: “valence” - corresponds to the type of emotion and represents how a human being feels (X axis); “arousal” - refers to emotion intensity and measures propensity to perform an action triggered by the emotional state (Y axis); “coping potential” - assesses the organism’s feeling of control over a given event (main diagonal); and “goal attainment degree” - weights the ease of achieving one or more goals (secondary diagonal)^(18)^.

The emotions analyzed in this study are based on the model proposed by Ekman (1973 and 2006), with the addition of the expression “Neutral” (as ground zero for emotions), as it is a more recent approach^(19)^.

**Figure 2:**
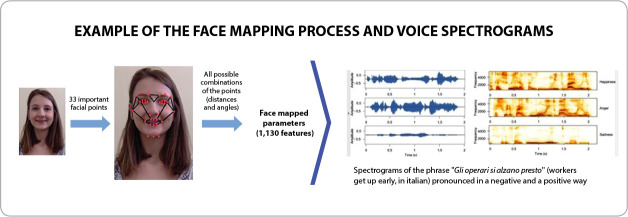
Face mapping and voice spectrograms

*Source: Mano et al. (face mapping)^(9)^ and Mano, Vasconcelos and Ueyama (voice spectrograms)^(21)^.*

For a better understanding, Figure 2 presents a compilation of processes of recognition and classification of emotion by face and tone of voice.

In the facial classification, a mapping process is carried out, considering 33 points obtained in the extraction of facial features. Dots are specific parts of the face, and together they uniquely identify an individual^(2)^.

Regarding tone of voice, classification was based on Mel Frequency Cepstral Coefficient (MFCC) and Log Energy ^(21)^. The difference between voice signals that express different emotions is mainly in the way the signal energy spectrum behaves. Thus, when comparing the frequency spectrum of a sentence pronounced in a negative way with the spectrum of the same sentence, expressed in a positive way, we will notice differences in energy spectral distribution^(21)^.

To enable this analysis, we used the Tone Analyzer software, which uses natural language processing and AI, focusing on identification and classification regarding emotional, linguistic and social tones contained in the text, based on work frequency assessment, a standard established and supported by the field theory of psychology^(22)^.

The use of AI has been used by researchers as a way to better understand the relationship between satisfaction, self-confidence and emotions that permeate nursing students as they experience a simulated environment^(9,19,23)^. It is believed that this method allows a closer study data analysis, which were collected on video with the use of camcorders, allowing to identify both faces and tone of voice and speech content.

The material collected then underwent two analytical processes: a content analysis, performed by the author-researcher and, concomitantly, another performed using AI software, through its developer, who is also a co-author of this work.

### Study setting

The study was conducted at a public university in Brasília, Federal District, Brazil. Maternal-child simulation is a curricular component of a nursing course at the higher education institution in question, and was carried out in the Simulation and Care Skills Laboratory both for practical activities and for summative assessment^(22)^. Data collection took place in a meeting room in one of the university’s buildings, away from the aforementioned laboratory, in order to guarantee the necessary privacy for carrying out the Focus Group (FG) technique. Participants in this room were the researcher as FG moderator, as she already had experience with the method, and two research assistants (volunteer graduate students - masters), to control the camcorders. The FG methodological design followed the same script of a study carried out with university students in Canada, in which the same technique was used in data collection^(24)^, listing guidelines for participant selection, collection and qualitative analysis^(25)^.

Neither the moderator nor the research assistants had any academic link with the study participants (undergraduate nursing students), but with the graduate studies at the aforementioned university.

### Data source

The population defined for this study included nursing students in the 7th semester who were taking the subject Comprehensive Care for Women and Children’s Health (CCWCH) (theoretical subject) concomitantly with Introduction to Practice Scenario 5 (practical in hospital and primary care services, with a focus on maternal-child health). Of the 46 students enrolled in the course, all were invited to participate in the research by the main author, in person, twenty days before a simulation activity was carried out in the theoretical subject, with the authorization of the course professors. The invitation consisted of an explanation of the research purpose, with clarifications about risks and benefits, the presence of research assistants and the signing of an Informed Consent Form and an Image and Sound Use Form. A total of 18 students did not wish to participate in the study, and therefore only 28 participants were included in the final sample.

A randomization was performed using the Flip application software by the call number corresponding to each participant. Thus, all students were allocated by groups and days of the CCWCH simulated activity, which took place before the FG data collection. Allocation took place over three days: day 1 (06/27/2019) - FG 1 and 2, with 4 students each; day 2 (06/28/2019); and day 3 (07/05/2019), with 2 FGs in each, with 5 students per group.

We excluded students who attended on the day of data collection with registration cancellation on the mentioned date, physical and emotional fatigue and lack of interest in participating in the activities. The answers were self-reported when students were questioned, before entering the room, where the FG would be held. However, there was no exclusion other than the 18 previously mentioned.

According to the CCWCH professors, cases created for the maternal-child clinical simulation activity, applied at the end of the semester, were related to the theory and practical experiences that students had throughout the semester: in health services and in the subject’s practical activities, in high-, mediumand low-complexity clinical cases, with low-, mediumand high-fidelity simulators and with standardized patients, to give realism to the simulated process.

### Data collection and organization

All FG sessions took place 10 minutes after students had completed the maternal-child clinical simulation activity. Those who had already agreed to participate in the study were led by a research assistant to the meeting room, located in the building next door, a room that resembled a study room rather than a classroom.

The collected data were recorded on two cameras owned by the researcher. After completing the FG sessions, the first researcher transcribed all speeches into a Word Office file for further analysis: content and AI. The recordings were downloaded and saved to the computer and archived in Drive, in Mp4format.

Clinical cases used in a CCWCH simulation activity were prepared by professors specializing in maternal-child matters and adjusted to the local reality. We used low-, mediumand high-fidelity simulators, in addition to patients standardized and previously trained by professors, to give realism to the simulated process. Standardized patients were subject monitors or graduate nurses, all with experience in the situations presented in the simulation’s clinical cases.

The simulated cases used were not previously validated for this activity. Not all professors had training/education by an accredited entity in simulation for teaching to implement it in health education, although they had previous experience. Assistants and scenario actors also had experience with simulation, knew what the goals of that experience were and knew the evaluative checklists and the expected step-by-step in each scenario.

We know from participants’ reports that the design adopted in CCWCH clinical simulation followed that recommended by the International Nursing Association of Clinical and Simulation Learning (INACSL)^(26)^ for good clinical simulation practices: pre-briefing, scenario, debriefing. On the first day, students faced two scenarios: the first was to assist a newborn with jaundice and a puerperal woman with difficulty for breastfeeding; the second was a prenatal care consultation (PC) to a pregnant woman and a growth and development consultation (GD) of a child of the pregnant woman’s cousin who accompanied her in PC consultation.

On the second day, cases were: about nursing care in the pre, trans and postoperative period of pregnant adolescents with pregnancy complications (pregnancy hidden from the mother); and about assistance to pregnant woman complaining of headache, dizziness and report of a seizure at home, culminating in the birth of a hypotonic newborn, with absent crying and spontaneous breathing. On the third day, clinical cases were: about pediatric patient safety (12 years) in the postoperative period of appendectomy surgery; and about childbirth and postpartum nursing care of pregnant women who arrive at the emergency room (ER) with dizziness, and, after childbirth, the newborn has an 1’7 and 5’8 APGAR.

### Methodological procedures

Using the FG technique^(24)^, each session followed the same guide-script (clarifications, opening, topic discussion and closing). The discussion was fostered by guiding questions of a structured script, with interconnected open-ended questions prepared by the researchers, to extract the values and meanings that students gave to the maternal-child clinical simulation experience as an educational strategy.

The questions were conducted by the main researcher, namely: how was the experience to you? Do you believe you were prepared for maternal-child simulation? In your opinion, did you gain knowledge and experience with the maternal-child simulation activity? Was there anything that would have been better for your learning if it had not occurred in mother-child simulation? How did the debriefing moment contribute to your academic experience? How did it contribute to your learning? What can you tell us about the debriefing moment?

During the sessions, some spontaneous questions arose to encourage a deeper understanding of the answers, feed discussion among participants and help them to describe points of view on the topic discussed, their perceptions and emotions, using, as a guide for this step, the practical guide tips for qualitative research^(25)^. The script developed for this study was not previously validated, but it is similar to the one adopted in a study with nursing students, which sought to understand students’ perception of psychological safety in the practice of simulation, in the sense of promoting a safe learning environment^(15)^.

Everyone had the opportunity to discuss their impressions on the subject, even if the subject had reached saturation. There was no return of transcripts to participants, because, at the moment of “closing”, a summary was made and it was verified if they wanted to rectify or ratify any speech. There were no requests for corrections. The FG sessions lasted about ninety minutes.

### Data analysis

The first step of data analysis involved content analysis, proposed by Bardin and widely used in the area of social, human and health knowledge, following text corpus systematic of pre-analysis, analysis and interpretation^(27)^, from speech transcription (text skimming, material preparation, material coding: with the choice of theme and context registration units; categorization, classification and data clustering by similarity)^(27)^. In the end, two categories emerged, “It was not easy, it was very stressful” and “Very valuable experience”.

In the second step, AI was used, choosing Scherer’s Circumplex Model^(1)^ as theoretical-methodological framework to analyze subjects’ emotional behavior. Data analysis, when submitted to AI, had the information from the footage ungrouped into “faces, voices and speech content”, analyzed by software: one mapped the facial points, considering 33 points and revealing the valences using the Circumplex Model; MFCC and Log Energy were used for voice analysis; and Tone Analyzer did natural language processing and AI, focusing on identifying and classifying the emotional, linguistic and social tone contained in the text.

## RESULTS

### First step: content analysis

Two categories of analysis emerged. The first category was called “It was not easy, it was very stressful”, through which participants made it clear that the simulation experience was full of feelings considered as “bad or negative”, not favoring learning and being summarized in the following statements:

*It was not easy, it was very stressful, it is still very stressful.* (D1S4)

*It’s really a bit stressful before, we get very anxious, but I believe that, despite all this, it’s a very rich experience for us.* [...] *I did not feel prepared at all because it generates anxiety, fear* [...]. (D1S3)

*I got a little lost, both in terms of the organization of the scenario itself and in terms of knowing what to do* [...]; *in this specific scenario of women’s and children’s health, we have no experience of simulation* [...]. (D2S3)

In the second category, called “Very valuable experience”, participants recognized that, even with difficulties, whether in the planning process, whether in the organization or even in the operationalization, maternal-child simulation was important for their own professional growth and maturation, summarized in the statements:

*For me, this is the most valuable moment of all, so we have the opportunity to reflect on everything that has been accomplished and everything that could be accomplished, together with professors’ knowledge.* (D1S1)

*Well, I think that simulation added a lot in the matter of relating the practice that we had seen, with the theory.* (D1S2)

*I found it a very valuable experience, I think it helps a lot in students’ growth.* (D1S1)

### Second step: analysis by Artificial Intelligence

In this step, FG session footage (image, audio and text) had a different treatment so that data could be analyzed by AI software. The components extracted from the FGs were the images of the face, the spectra of the recorded voice and the content spoken by each participant. Thus, in total, there were n= 23,888 analyzed faces; n= 47,766 energy frequency spectra for tone of voice; and n= 34,047 texts analyzed for speech description. The data comprise the sample of 28 nursing students who participated in the research.

**Figure 3:**
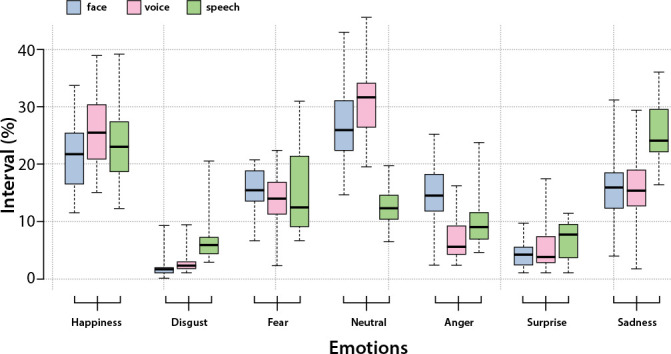
Variation of emotions observed in the three classifications among participants

Figure 3 represents the variation of emotions observed by quartiles, in the three classifications of the study. In this figure, there are seven boxplots.

**Figura 4:**
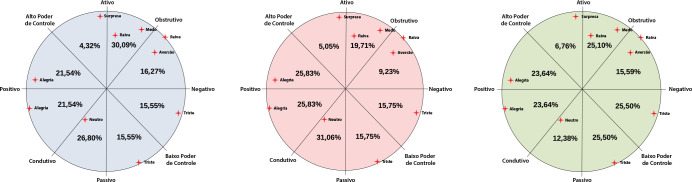
Modelo Circumplexo de Scherer com a distribuição, em porcentagem, das emoções que os participantes apresentaram, exposto nos eixos X, Y e nos eixos diagonais (principal e secundário)

Figure 4 shows the distribution of emotions in the Circumplex Model, revealing in percentage the emotions in each analyzed component: 1) classification by face (blue circle); 2) classification by tone of voice (pink circle); and 3) classification by speech description (green circle).

This graph focused on the axes with the highest percentage of emerging emotions, including how participants felt (X axis), the intensity of emotions felt (Y axis), the feeling of control in the face of the situation experienced (main diagonal axis) and the ease of achieving the teaching activity experienced objectives (secondary diagonal axis).

We noticed, however, that the emotional distribution was different between the face, voice and speech graphs. The emotions related to face analysis were mostly concentrated in the octant 1 (30.09%) (negative valence); those related to tone of voice analysis were concentrated in the octant 5 (31.06%) (positive valence); those pertinent to speech description analysis were concentrated in octants 3 and 4 (25.50%) (negative valence).

This allows us to state, according to this model, that, in facial analysis, students presented emotions related to a degree of negative valence, high degree of activity, medium power to control the situation and high degree of obstruction in task accomplishment. Thus, emotions such as anger, fear, tension, impatience, indignation, alarm, envy, among others, are linked to the characteristics of this octant.

In tone of voice analysis, students showed emotions related to a degree of positive valence, medium-high degree of passivity, medium power to control the situation and high degree of conduction in task accomplishment. This octant presents emotions such as neutral, empathy, polite, longing, peace, among others.

Finally, in speech description analysis, students showed emotions linked to a degree of negative valence, medium-high degree of passivity, low power to control the situation and medium-high degree of obstruction in task accomplishment. Emotions such as sadness, apathetic, worried, dissatisfied, disappointed, bored, among others, are linked to the characteristics of these two octants.

## DISCUSSION

Clinical simulation can be used in different contexts in teaching, combined with summative assessment, with grade attribution or only formative assessment, as a practice for training technical skills, nursing course screening, training of accidents with multiple victims, with students at any stage of graduation, or even in hospital accreditation and qualification of health professionals^(28-31)^.

Using AI, the Circumplex Model and emerging theories such as Cognitive Load Theory and Connectivism Theory, professors have a knowledge base that allows them to identify crucial points in students’ emotional learning process during simulation, in addition to enhancing these activities, in order to enrich and positively influence nurses’ training^(7,23,26,28-32)^.

It is important that the professors involved in this training have the ability to conduct simulation, especially debriefing, leading students to self-reflection and using effective and respectful communication, ensuring student engagement in the learning process. Thus, the opportunity to achieve the expected results in the simulation-based experiment^(26,31,33)^ is avoided.

Although students’ speeches in this study do not show the exact cause of their negative emotions, these may be linked to the simulation experience itself: because they do not have complete mastery of content (intrinsic cognitive load); because of lack of desired emotional security in the simulated environment (which comes from pre-briefing to debriefing - external cognitive load); because of fear of compromising teamwork; or because of fear of assessment, a result that is also present in other studies with clinical simulation in nursing ^(7,15,33-34)^.

Concisely, the results of this study converge with those of others, which highlight professional growth and maturation such as the development of self-confidence, communication skills, team interaction, leadership work, conflict management, reflection in action, learning through error, the ability to relate theory to practice, among other perceived gains^(8-9,30-33)^.

What drew attention in the results of AI was students’ emotional behavior. Such behavior highlights, in greater evidence, in the face, voice and speech content element octants, negative valence, i.e., negative emotions felt in the maternal-child simulation experience. On the arousal axis, medium-high degree of passivity stood out, i.e., the experience generated emotions that did not contribute to students having an expected active and/or proactive reaction in the scenario.

On the coping potential axis (main diagonal line), the medium power of control of the situation experienced in the simulated scenario prevailed. In the last axis (secondary diagonal), there was a higher prevalence for octants with a medium-high degree of obstruction in accomplishing the task faced in simulation.

For some reason, students, faced with simulation, have gone blank, which prevented them from acting and demonstrating in practice what they had studied or even carried out in health services in the practical subject throughout the academic semester. Professors should reflect on whether simulation, in addition to generating experience and positive emotions, can generate negative emotions and obstruct behavior or cognition, compromising student learning.

### Study limitations

Although the professors had more than five years of experience in conducting clinical simulation in the maternal-child area, only one was certified as an instructor in clinical simulation. There was not a mental health specialist on the team; there was no comparison group with standard teaching to measure possible differences related to emotions. Other limitations included: having been collected in a single moment; not having been previously screened to identify differences in participants’ personality that could influence emotional behavior in simulation; not having prior information about participants’ intellectual abilities; and not having been able to screen participants’ emotions and sex, since hormones influence individual responses and modulate human emotions.

### Contributions to teaching with simulation in maternal-child nursing

This study revealed positive and negative valence emotions experienced by nursing students after maternal-child simulation activities. However, it contributed to identify factors intrinsic to students inserted in the learning process, relevant for professors to be attentive and thus manage the emotions expressed.

## FINAL CONSIDERATIONS

The analyzes presented in this study reveal that, in the teaching-learning process in mother-child simulation, students oscillate between positive and negative emotions. It is observed, however, that simulation allows a previous experience of care practice, making use of articulation of theory with practice, preparing students for contexts that may be similar in their professional reality.

The information obtained from the study shows that the use of simulation is beneficial for students’ meaningful learning process, but reveals that it is necessary to carefully plan each step, in order to guarantee an experience in which cognitive load is allied to balanced emotional loads, allowing brain/students to learn new content, with feelings of positive valence, with high conductive and active power control.
